# Rapid propagation in vitro and accumulation of active substances of endangered *Dendrobium cariniferum* Rchb. f

**DOI:** 10.1080/21655979.2020.1739406

**Published:** 2020-03-14

**Authors:** Wei Lin, Jingjing Wang, Xiuming Xu, Yuhan Wu, Dongliang Qiu, Bizhu He, Surendra Sarsaiya, Xiaokai Ma, Jishuang Chen

**Affiliations:** a FAFU and UIUC-SIB Joint Center for Genomics and Biotechnology, the Key Laboratory of National Forestry and Grassland Administration for Orchid Conservation and Utilization (Fuzhou), College of Horticulture, College of Food Science, College of Forestry, Fujian Agriculture and Forestry University, Fuzhou, China; b Bioresource Institute for Healthy Utilization, Zunyi Medical University, Zunyi, China

**Keywords:** *Dendrobium cariniferum*, propagation in vitro, bioactive compounds, medium, tissue culture

## Abstract

*Dendrobium cariniferum* is a valuable ornamental and medicinal plant rich with polysaccharides, alkaloid, and other bioactive compounds, which are potential raw materials for pharmacological utilization. In this study, an efficient protocol for the rapid propagation of *D. cariniferum* was developed. By using the tissue culture protocol, the effects of pH, hormone combinations, temperatures, light intensity, culture time protocorm proliferation, seedlings rooting, and accumulation of biomass with bioactive compounds were investigated. The experiments showed that the medium [1/2 MS + activated carbon1.0 g/L+ agar strip 7.5 g/L + sucrose 25 g/L] effectively promoted the germination of *D. cariniferum* seeds. The optimal culture conditions were found at pH 5.7, temperature 23 ± 2°C, and light intensity of 1000 Lx in the protocorm proliferation stage. Adding 1.5 g/L peptone in the medium effectively promoted the seedling rooting. The optimal culture conditions for accumulation of bioactive compounds (polysaccharides and alkaloids) of seedlings were found at temperature of 25 ± 2°C, light intensity of 1500–2000 Lx after the 60-day (d). Our study constructed a rapid propagation system in vitro for *D. cariniferum*, as well as the methods for efficient accumulation of active substances in seedling culture, which will serve as guidance for industrial production of *D. cariniferum* seedlings for both medicinal raw materials and ornamental plants. In addition, our study provided a new idea that we can directly use the high bioactive compound seedlings to extract medicinal components in industry conditions without transferring to the field.

## Graphical Abstract

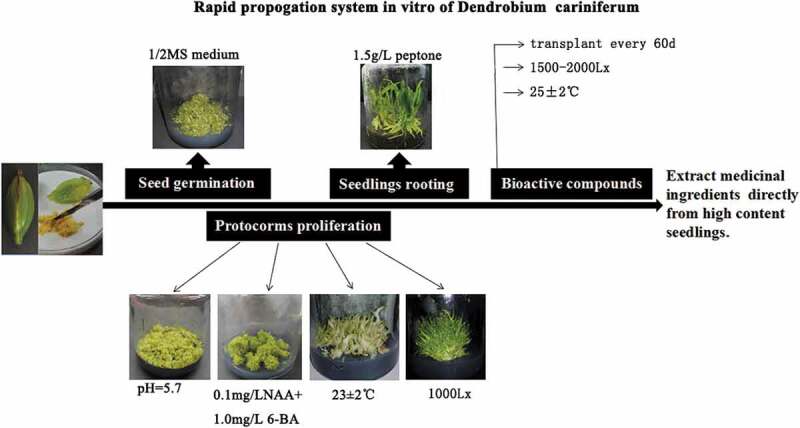

## 1. Introduction

*Dendrobium* is one of the largest genera in the Orchid family, with great diversity in floral characteristics and plant metabolic features, which is of considerable value to researchers, breeders, and biotechnologists [1]. *Dendrobium* species have high values in ornamental cultivation and medicinal unitization. There are several species that have been used in traditional medicine, such as *Dendrobium officinale* Kimura et Migo, *D. nobile* lindl, as well as *D. huoshanense. D. officinale* is a famous herb in traditional Chinese medicine, with *D. officinale* polysaccharides (DOP) and betaine as the main active ingredients, which exhibited a strong effect on antioxidant activity, leading to a significant antitumor effect [2]. *D. nobile* alkaloid (DNLA) was found to be able to improve insulin resistance and brain memory and recognition knowledge of dysfunction, showing anticancer and antileukemia activities [3,4]. *D. huoshanense* polysaccharide (DHP) is a harmless medicine which has resistance activities in liver damage and drug toxicity [5,6]. Except for those traditional medicines, a growing number of species in *Dendrobium* were also found to have potential novel application values in medicinal and ornamental unitization.

**Figure 1.**
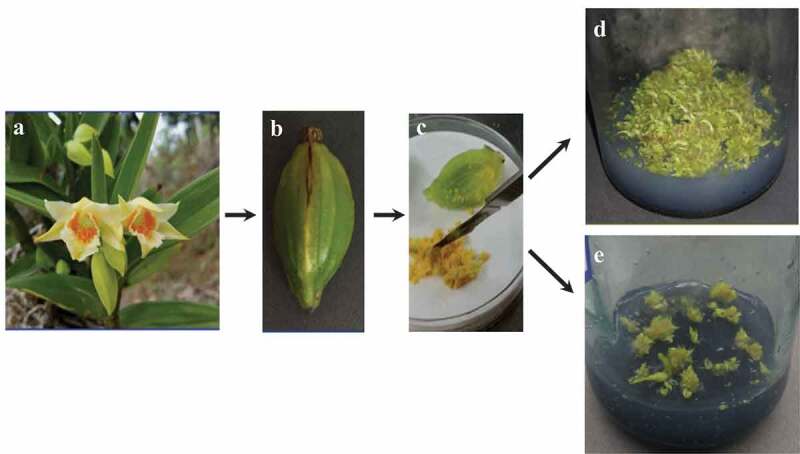
(a) Flowers of *Dendrobium cariniferum* after pollination. Fruit capsule (b) and seeds (c) of *D. cariniferum*. (d) Seed germination in one-half MS medium. (e) Seed germination in MS medium.

*D. carinferum* Rchb. f. (Figure 1(a)) is an endangered perennial herb with the genus *Dendrobium*, with its distribution mainly in southern Yunnan, China. It grows on the trunk and stone crevices of the warm and humid subtropical mountain areas. *D. carinferum* has bright orange-colored and fragrant flowers (Figure 1(a)) with theblooming period from March to April, with a high ornamental value. In recent years, Hao et al. [7] used the Gas chromatography–mass spectrometry (GC-MS) technique to identify the alkanes and esters chemicals in *D. carinferum* and showed that *D. carinferum* has a high percentage of phthalate dibutyl, significantly more than *D. fimbriatum* Hook and *D. chrysotoxum*. Lindl. Wang et al. [8] analyzed and identified the medicinal components of *the Dendrobium* plant and found that the effective chemical components of *D. carinferum* have an obvious therapeutic effect on eye diseases. However, there are few studies related to the protection and bioactive compounds of *D. cariniferum*. On the other hand, the continuous arbitrary mining and destruction of the ecological habitats are decreasing the wild resources of *D. cariniferum* [9].

The tissue culture has received greater attention for plant-specific bioactive compound production as well as seedling regeneration, which has very high application values in pharmaceutical, cosmetic, and horticulture plant industries, and the tissue culture has been widely used in the aspects of rapid propagation and wild resource protection of dendrobium [10]. This technology can not only promote the industrialization of plant propagation to meet the market demand, but also can quickly and directly provide raw materials which have high active substances for the medicinal industry. Our study used the tissue culture method along with the development of a rapid in vitro propagation system for efficient accumulation of bioactive compounds in seedling culture by the endangered *D. cariniferum*, which could be used as a valuable approach for the industrial production of *D. cariniferum* seedlings. In addition, the optimum conditions for the accumulation of active substances were studied, which not only provided a new idea for making the full use of potential bioactive compound seedlings but also laid a foundation for the research and development of medicinal active substances as well as regeneration of wild resources.

## 2. Materials and methods

### 2.1. Pre-processing for capsules and inoculation of seeds

The capsules of *D. cariniferum* which came from Xishuangbanna, Yunnan province, were selected as the materials (Figure 1(a,b)). The capsules chosen as experiment materials should be  robust, with no dehiscence and no diseases and which should have pollinated for 90 d. After collecting capsules, they were put into a plastic bag and stored at 4°C freezer for later use. The black patches on the surface of the capsule were scratched off carefully with a scalpel to clean the surface impurities. After cleaning the surface of capsules, they was placed in a beaker with water and soaked in a detergent for 5 min. Then, they were washed with sterile water for 3 times and then ransferred into a clean culture bottle for later use. The inoculation of *D. cariniferum* seeds was carried out under aseptic conditions. Before inoculation, the ultra-clean worktable needed to be sprayed with 75% alcohol and wiped clean. Place the prepared medium, operation instruments after high-temperature sterilization, and alcohol lamp on a bench-top, sterilized by UV for 30 min. A part of the bench-top was pushed open for 20-min ventilation. First, soak the capsule with 75% alcohol for 30 s, rinse with sterile water for two to three times, again soak with 5% NaClO for 10 min, and then rinse with sterile water for three to four times. Second, place the instruments in75% alcohol, and burn thoroughly on alcohol lamps to disinfect instruments. The sterilized capsule was then placed in a sterile petri dish with tweezers. Third, transfer the seeds from the capsule with tweezers and insertinto the medium. It was noted that the seeds in each bottle of the medium should not be overseeded. Finally, label them accordingly.

### 2.2. Methods

#### 2.2.1. Investigation of culture medium on seed germination

Seed germination of the medium was composed of one-half MS and MS separately, and then extra-activated carbon 1.0 g/L, agar strip 7.5 g/L, and sucrose 25 g/L were added to each of two mediums. Eight bottles were inoculated for each treatment. Each experiment was repeated three times. After inoculation, materials were kept in a dark environment for about 10-d cultivation, and then transferred to a light environment after the seeds germinated. The germination status of seeds was observed and recorded regularly.

#### 2.2.2. Investigation of factors affecting protocorm proliferation

In the stage of protocorm proliferation, there are four groups of treatments: protocorms were cultured in the medium of different pH, hormone combinations, temperature, and light intensity, which were designed to explore the optimal culture conditions for the protocorm proliferation of *D. cariniferum*. In addition, activated carbon 1.0 g/L, agar strip 7.5 g/L, and sucrose 25 g/L were added to the medium during these four experiments. After cultivating for the specified days, the growth status of protocorm and the fresh weight were observed and recorded, and the optimal pH value, hormone combinations, temperature, and light intensity of protocorm proliferation were explored.

##### 2.2.2.1. Treatments of different pH

Five groups of medium with different pH values (5.6, 5.7, 5.8, 5.9, and 6.0, Table S1) were settled for comparison after 45 d of growth.

##### 2.2.2.2. Treatments of different hormone combinations

Naphthalene acetic acid (NAA) concentrations were set as 0.1 mg/L and 0.5 mg/L, and 6-BA (benzyl adenine) concentrations as 1.0 mg/L and 2.0 mg/L. Six hormone combinations (A: 0.1 mg/L NAA+2.0 mg/L 6-BA; B: 0.5 mg/L NAA+2.0 mg/L 6-BA; C: 0.5 mg/L NAA; D: 0.1 mg/L NAA; E: 0.5 mg/L NAA+1.0 mg/L6-BA; F: 0.1 mg/L NAA+1.0 mg/L 6-BA, Table S2) were settled for comparison after cultivation for 60 d.

##### 2.2.2.3. Treatments of different temperatures

Three culture temperature (21 ± 2°C, 23 ± 2°C, and 25 ± 2°C, Table S3) were settled for comparison after cultivation for 90 d.

##### 2.2.2.4. Treatments of different light intensities

The light intensity was set as 0 Lx, 500 Lx, 1000 Lx, 1500 Lx, 2000 Lx, and 2500 Lx (Table S4), respectively, for comparison after cultivation for 90 d.

#### 2.2.3. Investigation of the factors affecting seedling rooting

Three concentrations of peptone were chosen, respectively, as 0.5 g/L, 1.0 g/L, and 1.5 g/L for comparison (Table S5). After 90-d cultivation, the growth status of the protocorm and the fresh weight was observed and recorded to investigate the optimal peptone addition concentration for seedling rooting.

#### 2.2.4. Investigation of factors affecting the accumulation of active substances in seedling culture

Sample preparation: 6-month old seedlings were collected from different cultivation conditions. The materials were dried in a drying box after cleaning the root medium for 30 min under 121°C and full drying under 85°C. The dried materials were grinded to powder and packed in a self-sealing bag with an appropriate label. The content of polysaccharide and alkaloid in different culture times (60 d, 90 d, Table S6), culture temperature (21 ± 2°C, 23 ± 2°C, 25 ± 2°C, and 27 ± 2°C, Table S7), as well as different light intensities (0–500 Lx, 500–1000 Lx, 1000–1500 Lx, 1500–2000 Lx, and 2000–2500 Lx, Table S8) were measured and compared.

##### 2.2.4.1. Determination of polysaccharide content in cultured seedlings

The preparation of the standard curve of polysaccharides (Fig S1-A) was carried out by referring to the improved methods. The sample of sucrose was precisely weighed and dried at 100 mg, and the volume was fixed to 1000 mL with deionized water. 0.05, 0.1, 0.2, 0.4, 0.6, and 0.8 mL of double-distilled water was added to 2 mL and then with 1 mL of 9% phenol solution in sequence and shaken well; then, 5 mL of concentrated sulfuric acid was added and shaken well. After cooling to room temperature, the absorbance value was measured by colorimetry at a wavelength of 485 nm in the blank group as control.

Extraction and determination of polysaccharides from *D. cariniferum*: 0.03 g of plantlet powder was precisely weighed and placed in a 2-mL centrifuge tube. Then, 1 mL of double-steamed water was added. After boiling in a water bath for 30 min, it was placed in a TdL-40B centrifuge (Shanghai Anting Flying Pigeon Equipment Co., Ltd., Shanghai, China). 1 mL of double-steamed water was added to the precipitate in the centrifuge tube, and centrifugation was done for 5 min and then the supernatant was taken. The residue was dissolved and washed again with double-steamed water and centrifuged for 5 min before taking the supernatant. The supernatant was fixed with double-steamed water to 10 mL; 5 mL of petroleum ether was added to the solution, and the desktop constant temperature oscillator (Wuxi Longtai Chemical Machinery Equipment Co., Ltd., China) was used to vibrate at room temperature for 10 min at 200 rpm/min, and then the same was stratified statically, and the upper layer solution was discarded. After static stratification, the upper solution was transferred into the 20-mL centrifuge tube by a pipet-holding gun . After the 30 minutes, the colour was analysed at 485 nm by using the visible spectrophotometer (Beijing Puri General Instrument Co., Ltd., Beijing, China). The method of the standard curve was adopted to determine the content of polysaccharides (Table S9), and the content of polysaccharides in samples was calculated according to the following formula. Content of polysaccharide (%) = (sugar content found on the standard curve of polysaccharide/volume of sample solution used for determination) × volume of extracted liquid × (dilution/sample weight) ×10^6^ × 100%

##### 2.2.4.2. Determination of alkaloid content in cultured seedlings

Preparation of the standard alkaloid curve (Fig S1-B): trigonelline 9.35 mg was weighed in a 50-mL volumetric flask, dissolved in methanol, and fixed in volume, and 0.187 mg/mL control solution was obtained. It was stored at low temperature for later use. 0.5 mL, 1 mL, 1.5 mL, 2 mL, and 2.5 mL were absorbed into the liquid separation funnel, followed by 7 mL buffer of sodium citrate with pH 5.6 and 2 mL of bromothymol blue. Chloroform was extracted for 3 times, each time 15 mL, each time oscillating for 60 s, and then let stand for 30 min before filtration. Extraction and determination of *D. cariniferum* alkaloids: 0.1 g of dendrobium powder was weighed, placed in a 10-mL volumetric bottle, and 8 mL of methanol was added for 2 h after using type KH-600 KDE High-power CNC ultrasonic cleaner (Wuxi Longtai Chemical Machinery Co., Ltd., China) (30°C, 250 w, 55 khz). After filter the content, 0.5 mL of methanol extract, 7 mL of sodium citrate buffer with pH 5.6, and 2 mL of bromothymol blue were placed in a separating funnel and were extracted with chloroform for 3 times, each time 7.5 mL, each time shaking for 60 s, and then allowed to stand for 30 min before filtration. The chloroform layer was taken and combined with the extraction fluid. 0.5 mL of the extraction fluid was taken, and 4.5 mL of methanol was added for 10× dilution (Table S10). According to the following formula:

Alkaloid content (%) = (C*N1*V1*V3)/V2

C (mg/ml): the concentration of the diluted extract; N1: dilution factor of the extract; V1 (mL): the extracted liquid product; V2 (mL): the liquid product for test; V3 is the total volume of the extract.

#### 2.2.5. Data analysis

IBM SPSS Statistics 23.0 software was used for statistical analysis. Independent sample *t*-test was used for comparing the different significance of the proliferation rate (PR) and accumulation of bioactive substances under different conditions and factors. GraphPad Prism 5.01 software was used to make bar charts.

## 3. Results

### 3.1. Effect of basic medium on seed germination

After artificial pollination for 90 d, the capsules of *D. cariniferum* (Figure 1(b)) were collected. The seeds were cut (Figure 1(c)) and put into one-half MS and MS medium separately. Through comparing with the seed germination status in these two basic media, we discovered that germination status has a significant difference under one-half MS and MS basic medium after cultivated for 30 d. The seeds in one-half MS basic medium germinated faster, differentiated loosely, and showed bright green color. The seed in MS (Figure 1(e)) basic medium germinated slowly and showed yellowish-green color. Therefore, one-half MS (Figure 1(d)) basic medium is more suitable for the germination culture of *D. cariniferum* seeds.

### 3.2. Effects of different factors for proliferation of protocorm

#### 3.2.1. Effect of medium pH value on proliferation of protocorm

**Figure 2.**
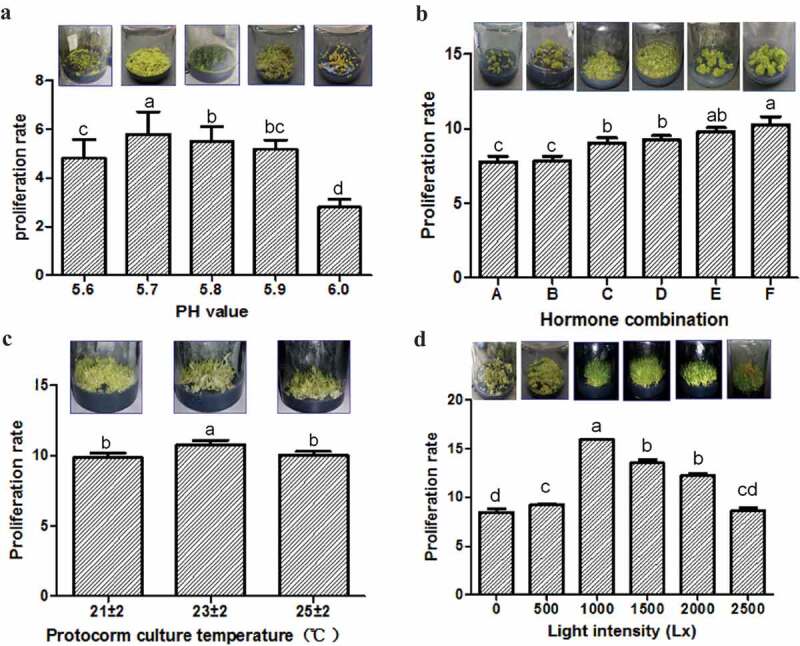
Effects of different factors on the proliferation of protocorms. (a) different medium pH; (b) different hormone combinations (A: 0.1 mg/L NAA+2.0 mg/L 6-BA; B: 0.5 mg/L NAA+2.0 mg/L 6-BA; C: 0.5 mg/L NAA; D: 0.1 mg/L NAA; E: 0.5 mg/L NAA+1.0 mg/L 6-BA; F: 0.1 mg/L NAA+1.0 mg/L 6-BA); (c) different temperatures; (d) different light intensities. The proliferation rate = the fresh weight after protocorm proliferation/fresh weight before protocorm inoculation. *Significance was determined by ANOVA (the same letter marks mean the difference was not significant: *P* ≥ 0.05, while different letters mean significant difference: *P* < 0.05).

The effect of medium pH (5.6, 5.7, 5.8, 5.9, and 6.0) was tested on the protocorm proliferation. The status and PR of protocorms varied with different medium pH. The protocorm presented the fresh olivine color, sprouted and differentiated quickly under pH 5.7 cultivation conditions. However, they sprouted and differentiated more slowly and showed a green color under pH 5.8 and a yellow color under pH 5.9 (Figure 2(a)). The PR was the highest at medium pH 5.7 (PR value = 5.8 ± 0.92, Table S1) than the others (independent sample *t*-test, *P* < 0.05, Figure 2(a), Table S1). The results, therefore, show that the optimal culture medium pH for *D. cariniferum* protocorm proliferation is 5.7.

#### 3.2.2. Effects of different hormone combinations on protocorm proliferation

Six different combinations of hormones (NAA 0.1 mg/L + 6-BA 1.0 mg/L, NAA 0.1 mg/L + 6-BA 2.0 mg/L, NAA 0.5 mg/L + 6-BA 1.0 mg/L, NAA0.5 mg/L + 6-BA2.0 mg/L, NAA 0.1 mg/L, and NAA 0.5 mg/L) were used to test the effects of hormone combinations on the protocorm proliferation. The performance and PR of protocorms showed differences under different hormone combinations. The protocorm presented with  a green color, sprouted, and differentiated quickly after adding (0.1 mg/L NAA+1.0 mg/L 6-BA) to the medium. While they sprouted and differentiated slower and showed a lighter green color under the other five hormone combinations (Figure 2(b)).The PR value is the highest at (0.1 mg/L NAA+1.0 mg/L 6-BA) hormone combination (PR value = 10.27 ± 0.52, Table S2) than the others (independent sample *t*-test, *P* < 0.05, Figure 2(b), and Table S2). The results finally showed the optimal combination of hormone for proliferation is the combination of (0.1 mg/L NAA+1.0 mg/L6-BA).

#### 3.2.3. Effects of different culture temperatures on protocorm proliferation

In order to test the effect of culture temperature on the protocorm proliferation, three groups of temperatures (21 ± 2°C, 23 ± 2°C, 25 ± 2°C) were used for comparison. Different culture temperatures have different status and PR of protocorms. The protocorm sprouted and differentiated quickly under 23 ± 2°C cultivation conditions. However, they sprouted and differentiated slower and showed a pale green color under 21 ± 2°C and a light green color under 25 ± 2°C (Figure 2(c)). The PR was the highest at 23 ± 2°C (PR = 10.75 ± 0.33, Table S3) than the others (independent sample *t*-test, *P* < 0.05, Figure 2(c), and Table S3). The result indicates the optimum culture temperature for *D. cariniferum* protocorm proliferation is 23 ± 2°C.

#### 3.2.4. Effects of light intensity on protocorm proliferation

Six different levels of light intensity (0 Lx, 500 Lx, 1000 Lx, 1500 Lx, 2000 Lx, and 2500 Lx) were set to test on the protocorm proliferation. The status and PR of protocorms were influenced by different light intensities. The protocorm presented with a bright green color, sprouted, and differentiated fastest under 1000 Lx cultivation conditions. However, the protocorm sprouted and differentiated more slowly and showed a light green color under 0 Lx, with pale green color under 500 Lx, and green color under 1500 Lx and 2000 Lx, as well as a brown color under 2500 Lx (Figure 2(d)). The PR is the highest at 1000 Lx (PR = 15.92 ± 0.06, Table S4) than the others (independent sample *t*-test, *P *< 0.05, Figure 2(d), Table S4). The result, therefore, shows the optimum light intensity for *D. cariniferum* protocorm proliferation is 1000 Lx intensity.

### 3.3. Effects of peptone concentration on seedling rooting

**Figure 3.**
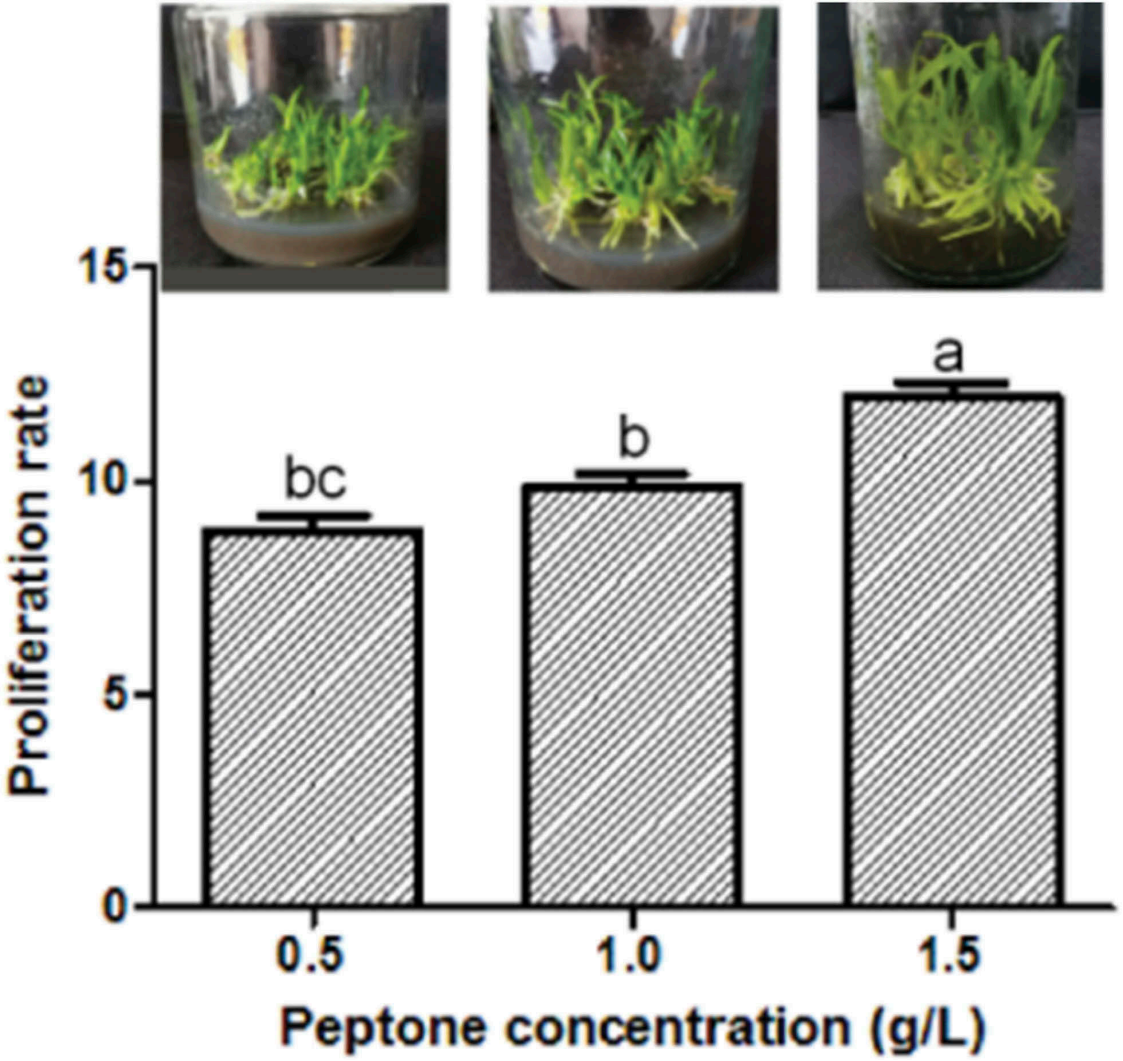
Effects of different concentrations of peptone on seedling rooting. *Significance was determined by ANOVA (the same letter marks mean the difference was not significant: *P* ≥ 0.05, while the different letters mean significant difference: *P* < 0.05).

Three groups of peptone concentration (0.5 g/L, 1.0 g/L, and 1.5 g/L) were used to test the PR of protocorms. The protocorm performance and the PPR varied when using different peptone concentrations. The protocorms generate the thickest seedlings and grow fastest under 1.5 g/L peptone medium. However, they grow more slower and showthinner seedlings under 0.5 g/L and 1.0 g/L (Figure 3). The PR was the highest at 1.5 g/L (PR = 10.01 ± 0.28, Table S5) than the others (independent sample *t*-test, *P *< 0.05, Figure 3, Table S5). The result indicated that the optimum peptone concentration for seedling rooting of *D. cariniferum* was 1.5 g/L.

### 3.4. Influence of factors for accumulation of active substances in seedling culture

#### 3.4.1. Influence of culture time on the accumulation of active substances in cultured seedlings

**Figure 4.**
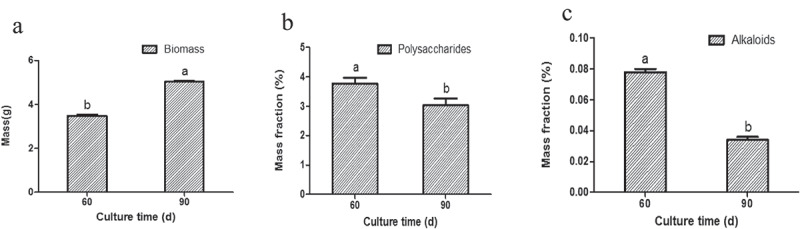
The influence of different culture time on the accumulation of biomass (a), polysaccharides (b), as well as alkaloids (c) in seedling culture. *Significance was determined by ANOVA (the same letter marks mean the difference was not significant: *P* ≥ 0.05, while the different letters mean significant difference: *P* < 0.05).

The effect of different culture time (60 d, 90 d) was tested on the seedling culture stage. The accumulation of biomass, polysaccharides, and alkaloids of the seedlings varied with different culture times. The seedlings showed an average of 3.48 g biomass, 3.763% polysaccharides, and 0.078% alkaloids when they were cultured 60 d. However, when the seedlings were cultured 90 d, they showed an average of 5.04 g biomass (Figure 4(a)), 3.027% polysaccharides (Figure 4(b)), and 0.034% alkaloids (Table S6). Although the culture time exceeded 60 d, the biomass increased (independent sample *t*-test, *P *< 0.05, Figure 4(a)) and the accumulated compounds decreased (independent sample *t*-test, *P *< 0.05, Figure 4(b,c)). The results indicated that the optimum culture time for seedlings to accumulate bioactive compounds was found at 60 d.

#### 3.4.2. Influence of temperature on the accumulation of active substances in cultured seedlings

**Figure 5.**
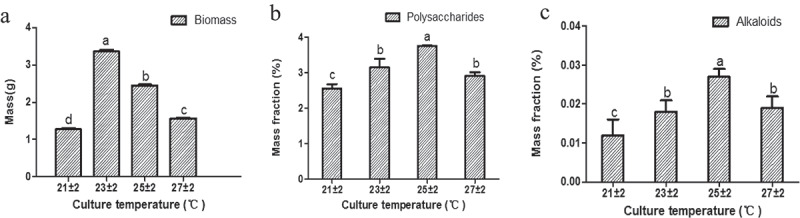
The influence of different degrees of culture temperature on the accumulation of biomass (a), polysaccharides (b), and alkaloids (c) in seedling culture. *Significance was determined by ANOVA (the same letter marks mean the difference was not significant: *P* ≥ 0.05, while the different letters mean significant difference: *P* < 0.05).

Four different levels of temperature (21 ± 2°C, 23 ± 2°C, 25 ± 2°C, and 27 ± 2°C) were settled to test the accumulation of the biomass, polysaccharides, and alkaloids of cultural seedlings. The biomass has the highest content under 23 ± 2°C (biomass = 3.36 g, Figure 5(a) and Table S7) than the others (independent sample *t*-test, *P *< 0.05, Figure 4(b), and Table S7). However, the polysaccharides (Figure 5(b)) and alkaloids (Figure 5(c)) both have the highest content under 25 ± 2°C (polysaccharides = 3.756%, alkaloids = 0.027%, Table S7) than the others (independent sample *t*-test, *P* < 0.05, Figure 4(b), and Table S7). The results, therefore, showed that the optimum temperature range for biomass accumulation was found at 23 ± 2°C, while 25 ± 2°C was the optimum temperature range for the accumulation of the bioactive compounds.

#### 3.4.3. Effects of light intensity on the accumulation of bioactive compounds in cultured seedlings

**Figure 6.**
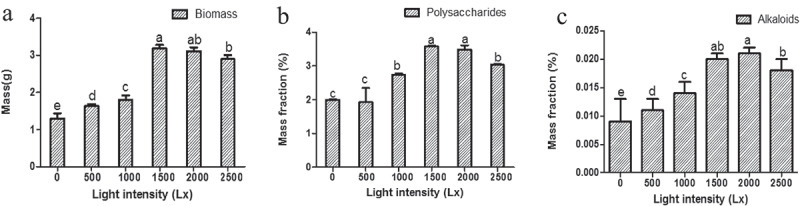
The influence of different levels of light intensity on the accumulation of biomass (a), polysaccharides (b), and alkaloids (c) in seedling culture. *Significance was determined by ANOVA (the same letter marks mean the difference was not significant: *P* ≥ 0.05, while the different letters mean significant difference: *P* < 0.05).

Six ranges of light intensity (0–500 Lx, 500–1000 Lx, 1000–1500 Lx, 1500–2000 Lx, and 2000–2500 Lx) were used to test the accumulation of the biomass, polysaccharides, and alkaloids of the cultural seedlings. Both biomass and bioactive compounds of seedling presented the highest level under 1500–2000 Lx light intensity culture condition (Figure 4(c)). However, they have lower biomass and lower levels of active substances under the other four light intensity range. The content of biomass (Figure 6(a)), polysaccharides (Figure 6(b)), and alkaloids (Figure 6(c)), all presented the highest level under 1500–2000 Lx (Biomass = 3.15 g, Polysaccharides = 3.532%, alkaloids = 0.021%, Table S8) than the others (independent sample *t*-test, *P* < 0.05, Figure 4(c) and Table S8). Therefore, the results showed that 1500 ~ 2000 Lx was the optimum range of light intensity for both seedling growth and accumulation of active compounds.

The one-half MS basic medium was suitable for seed germination. The optimal culture conditions are medium pH value at 5.7, temperature of 23 ± 2°C, light intensity of 1000 Lx, and hormone combinations [0.1 mg/L NAA+1.0 mg/L 6-BA] at the protocorm proliferation stage. Adding 1.5 g/L peptone to the medium effectively promoted the seedlings rooting. At the accumulation stage of bioactive compounds, the optimal culture conditions were found at a temperature range of 25 ± 2°C and a light intensity of 1500–2000 Lx. After 60 d of tissue culture, the seedlings need to be transferred in time. This culture system effectively promoted the rapid propagation in vitro and accumulation of bioactive compounds of *D. cariniferum.*

## 4. Discussion

### 4.1. Selection of medium for seed germination

In the stage of *D. cariniferum* seed germination, the culture medium of (one-half MS + activated carbon 1.0 g/L + agar article 7.5 g/L + sucrose 25 g/L) effectively promoted the germination of seeds, which was obviously better than the MS medium. The results indicated that the germination of *D. cariniferum* seeds required a lower salt concentration; too high or too low inorganic salt concentration are not beneficial for seed germination. The MS medium is rich in inorganic salts, especially ammonium nitrate, potassium ion, and ammonium ions, which is not suitable for seed germination. In addition, from the perspective of economy, the cost of one-half MS basic medium was relatively low. Therefore, one-half MS medium was selected as the basic medium for *D. carniferum* seed germination, which was found to be more economical and efficient. Chen et al. [10] proposed that the effect of one-half MS basic medium on seed germination was better than that of MS basic medium at the seed germination stage when they studied the effect of different mediums on aseptic seed germination of hybrid capsule for species pH.

### 4.2. Culture conditions for protocorm proliferation

At a medium pH value of 5.7, the protocorm proliferated and differentiated faster and loosely, which should be regarded as the optimum medium pH value for *D. cariniferum* protocorm proliferation stage. The experiments show that *D. cariniferum* seeds were more suitable to the acidic medium condition. Moreover, the result shows that 23 ± 2°C was the optimum temperature range for protocorm proliferation. Too low or too high temperature will have disadvantages on the quality of culture seedlings for *D. cariniferum* [11]. We found that the enzyme activity of cultured seedlings was possibly the main factor to influence this process. The intensity of light has a great influence on the growth of cultured seedlings as shown in *Dendrobium* and *Oncidium* [12]. We discovered that the roots of cultured seedlings differentiated more quickly, and the color of bud and stem becomes emerald green under strong light intensity. Conversely, the length of the cultured seedlings was long, but the resistance of seedlings was weak under low light intensity conditions. In this condition, some seedlings turn yellow, which was similar to the cultivation of *Cymbidium japonicus* [13]. Therefore, the optimal light intensity for protocorm proliferation of *D. cariniferum* was set to 1000 Lx. In addition, the combination of medium (0.1 mg/L NAA+1.0 mg/L 6-BA) was found to be the optimal hormone combination for protocorm proliferation. Similarly, the study on *Cymbidium hybridum* by Zhang et al. [14] believed that when the concentration ratio of NAA and 6-BA was 1:5, the differentiation and induction rate of multiple shoots for the protocorm was the highest.

### 4.3. Selection of peptone concentration at the seedling rooting

Peptone is a light-yellow powder hydrolyzed with acid or protease which is rich in various nutrients, such as nitrogen and carbon source [15]. Adding a suitable concentration of peptone to the medium effectively promoted the seedling rooting. Han et al. [16] showed that tryptone was beneficial to the proliferation of *Cymbidum* rhizomes. Our experiment concluded that the addition of 1.5 g/L peptone in the medium effectively promoted the rooting of *D. cariniferum* seedlings, which can be widely used for *D. cariniferum* seedling production.

### 4.4. Selection of cultivation conditions for the accumulation of active substances in seedling culture

The yield of *D. cariniferum* was determined by the biomass and the content of active substances. With the extension of time, the biomass was gradually increased; however, the active substances decreased, which is also found in a previous study [17]. In order to prevent the accumulation of active substances from being affected by nutrient deficiency in the medium at a later stage and ensure the normal accumulation of active substances, seedlings were transferred at every 60 d. Therefore, properly controlling the growth rate in a certain range was beneficial to the accumulation of active substances in seedling culture. In addition, under the light intensity of 1500 ~ 2000 Lx, the growth performance of cultured seedlings was the best, the overintense light and darkness had adverse effects on the seedlings, which indicated that the seedlings’ cultivation of *D. cariniferum* has strict requirements for the light intensity. Therefore, special attention should be paid to regulate the light intensity at the seedling stage [18–23].

Finally, the cultivation temperature could lead to the change of enzyme activity in seedlings. Our research concluded that temperature (23 ± 2°C) for cultivating seedlings will consume a large amount of material in the growth, which affected the accumulation of active material. Therefore, in order to ensure the normal accumulation of active substance, the temperature could set between range from 23-25 ± 2°C. Our results showed the seedlings of *D. cariniferum* were sensitive to the temperature; if the cultivation temperature is not suitable, it could lead to the change of enzyme activity of seedlings. The experimental result showed that the biomass accumulated at the highest amount under 23 ± 2°C culture temperature. However, the nutrition of seedlings was consumed for growth, which resulted in few bioactive compounds accumulated. While under 25 ± 2°C culture temperature, the seedlings grew slower, but bioactive compound accumulation increased with the highest level compared with other cultivation temperature.

## 5. Conclusions

Our study used the tissue culture process for developing a rapid propagation system in vitro and the methods for efficient accumulation of bioactive compounds in seedling culture for the endangered *D. cariniferum*. In addition, the optimum conditions for the accumulation of bioactive compounds in seedling culture were analysed through the development of a rapid in vitro propagation system. Our study provided a new idea that cultivated seedlings that containhigh active substances can be directly used to extract medicinal raw materials to save the process of transferring to the field growth.

## Highlights

- Developed a rapid propagation system for accumulation of bioactive compounds by *D. cariniferum*;
- Optimal conditions found at a temperature of 25±2°C, light intensity of 1500-2000 Lx for 60 days;
- Provided a new idea for direct extraction of medicinal components using seedlings;
