# Supplementary material for: Rapid propagation in vitro and accumulation of active substances of endangered Dendrobium cariniferum Rchb. f
Source: Bioengineered. 2020 Mar 14;11(1):386–96. doi: 10.1080/21655979.2020.1739406 (PMC7161565; doi:10.1080/21655979.2020.1739406)
Supplement: Supplemental Material [file kbie-11-01-1739406-s001.docx]

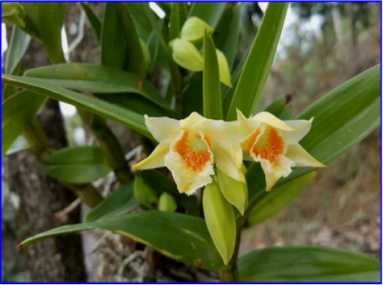

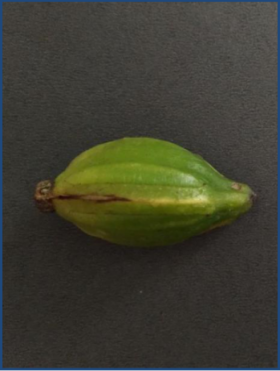

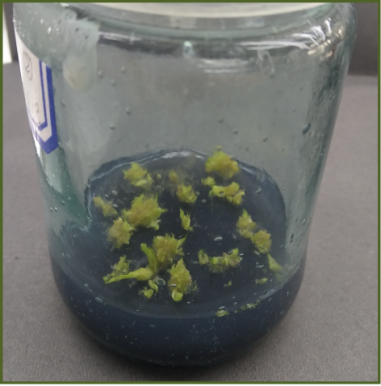

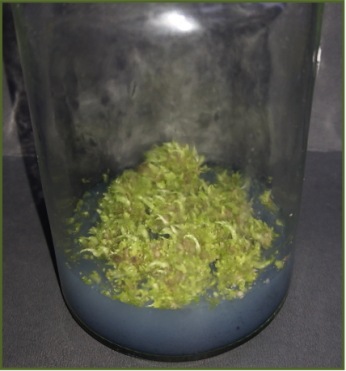

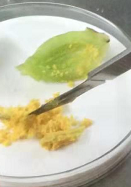


**A**

**E**

**D**

**C**

**B**

**Fig 1.** (A) Flowers of *Dendrobium cariniferum* after pollination. Fruit capsule (B) and Seeds (C) of *D. cariniferum*. (D) Seeds germination in ½ MS medium. (E) Seeds germination in MS medium.


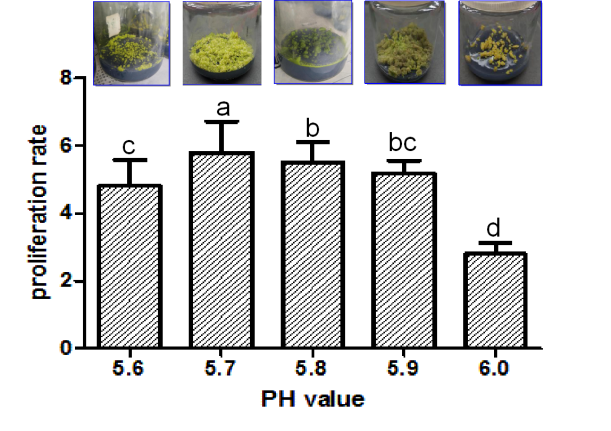

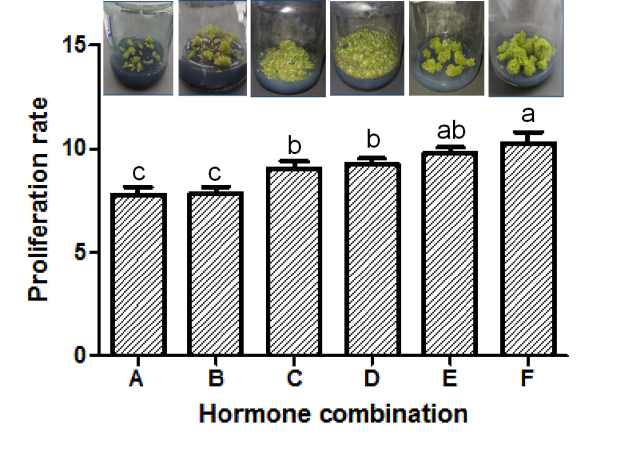

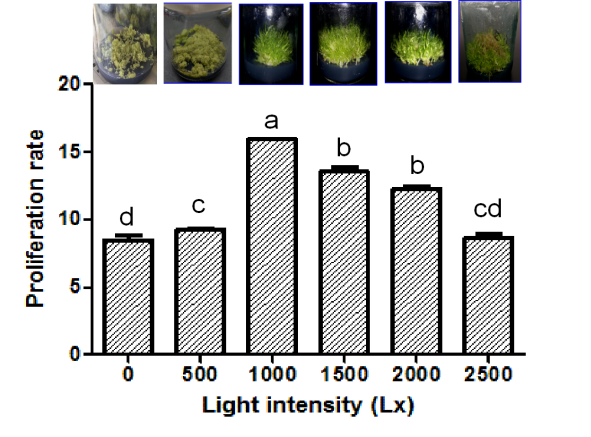

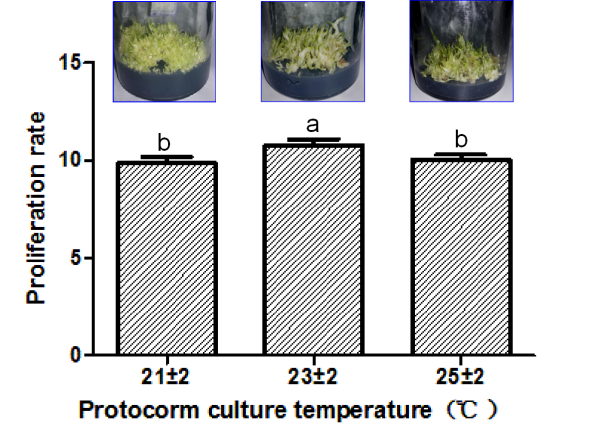


**A**

**C**

**D**

**B**

**Fig 2.** Effects of different factors on the proliferation of protocorms. (A) different medium PH; (B) different hormone combinations (A: 0.1 mg/L NAA+2.0 mg/L 6-BA; B: 0.5 mg/L NAA+2.0 mg/L 6-BA; C: 0.5 mg/L NAA; D: 0.1 mg/L NAA; E: 0.5 mg/L NAA+1.0 mg/L 6-BA; F: 0.1 mg/L NAA+1.0 mg/L 6-BA). (C) different temperature. (D) different light intensity. The proliferation rate= the fresh weight after protocorm proliferation/fresh weight before protocorm inoculation.

*Significance was determinated by ANOVA (the same letter mark means the difference was not significant: p>=0.05, while the different letter means significant difference: p<0.05).


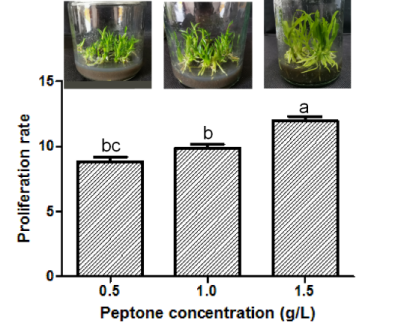


**Fig 3.** Effects of different concentrations of peptone on seedling rooting.

*Significance was determinated by ANOVA (the same letter mark means the difference was not significant: p>=0.05, while the different letter means significant difference: p<0.05).

A

C

B


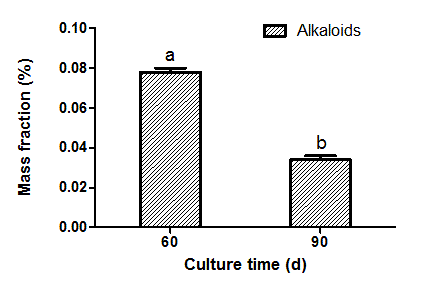

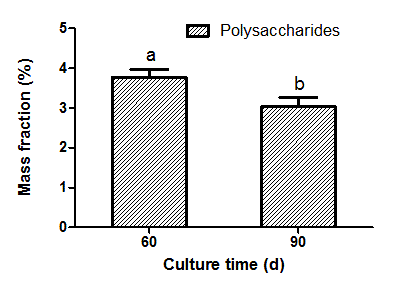

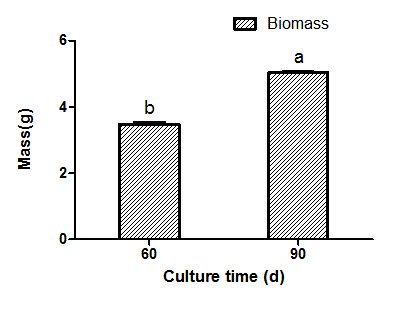


**Fig 4.** The influence of different culture time on the accumulation of biomass (A), polysaccharides (B), as well as alkaloids (C) in seedling culture.

*Significance was determinated by ANOVA (the same letter mark means the difference was not significant: p>=0.05, while the different letter means significant difference: p<0.05).


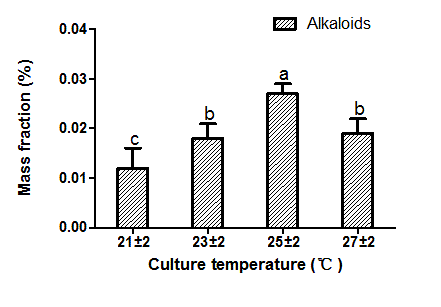

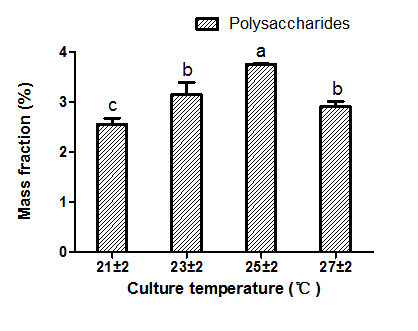

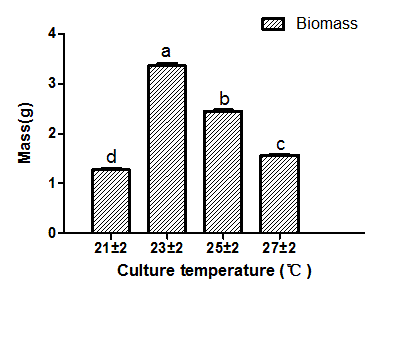


A

B

C

**Fig 5.** The influence of different degrees of culture temperature on the accumulation of biomass (A), polysaccharides (B), and alkaloids (C) in seedling culture.

*Significance was determinated by ANOVA (the same letter mark means the difference was not significant: p>=0.05, while the different letter means significant difference: p<0.05).


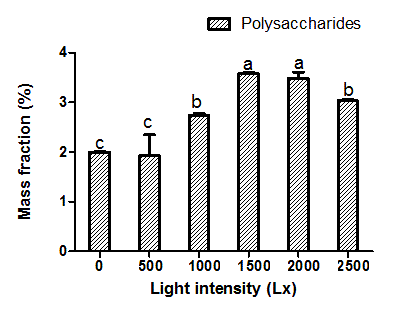

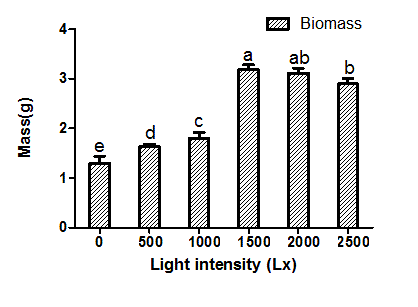

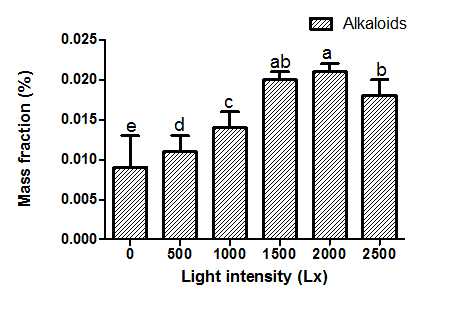


B

C

A

**Fig 6.** The influence of different levels of light intensity on the accumulation of biomass (A), polysaccharides (B), and alkaloids (C) in seedling culture.

*Significance was determinated by ANOVA (the same letter mark means the difference was not significant: p>=0.05, while the different letter means significant difference: p<0.05).
